# Functional insights to the development of bioactive material for combating bacterial infections

**DOI:** 10.3389/fbioe.2023.1186637

**Published:** 2023-04-21

**Authors:** Duoyang Fan, Xiaohui Liu, Yueming Ren, Shuaige Bai, Yanbing Li, Ziheng Luo, Jie Dong, Fei Chen, Wenbin Zeng

**Affiliations:** ^1^ Xiangya School of Pharmaceutical Sciences, Central South University, Changsha, China; ^2^ Hunan Key Laboratory of Diagnostic and Therapeutic Drug Research for Chronic Diseases, Changsha, China; ^3^ Xiangya Hospital, Central South University, Changsha, China

**Keywords:** antibacterial, multidrug-resistant bacterial biofilm, theranostics, nanomaterials, cationic polymers

## Abstract

The emergence of antibiotic-resistant “superbugs” poses a serious threat to human health. Nanomaterials and cationic polymers have shown unprecedented advantages as effective antimicrobial therapies due to their flexibility and ability to interact with biological macromolecules. They can incorporate a variety of antimicrobial substances, achieving multifunctional effects without easily developing drug resistance. Herein, this article discusses recent advances in cationic polymers and nano-antibacterial materials, including material options, fabrication techniques, structural characteristics, and activity performance, with a focus on their fundamental active elements.

## 1 Introduction

Bacterial infection has always been a major threat to human life and a serious global challenge (Rasko and Sperandio, 2010). The death cases caused by long-term chronic bacterial infection are increasing in clinical treatment. In addition, severe acute bacterial infection can lead to the failure of surgery, organ transplantation, and implantation of medical materials. With the potential risk of sepsis and even death, which has become a huge clinical hidden trouble (Poole, 2011). According to the World Health Organization (WHO) statistics, at least 700,000 people die from bacterial infections every year in the world. If no effective measures are taken, the number of people who suffer from bacterial infections will reach 10 million by 2050, causing a cumulative economic loss of more than 100 trillion dollars (Gupta et al., 2019). As one of the major advances in medicine in the last century, antibiotics can effectively kill bacteria and have great clinical applications. However, the long-term and repeated use of antibiotics have led to the emergence of multi-resistant superbugs, making it difficult for traditional antibiotics to work (Blair et al., 2015). In addition, bacteria form a protective biofilm during reproduction, which protects them from the harsh environment or drugs and effectively inhibits antibiotic penetration (Alfa, 2019). Clinical related problems caused by bacterial infections are becoming increasingly serious. Unfortunately, since the era of antibiotic resistance began in 2000, there has been no significant progress in the development of new antibiotic drugs based on traditional antibiotic antibacterial mechanisms (Tang et al., 2014). According to research, on the one hand, the emergence of bacterial resistance may be the result of the interaction of multiple drug resistance mechanisms. Due to the lack of understanding of the most important mechanism—drug efflux mechanism, developing antibiotics with appropriate structures is a huge challenge. On the other hand, the long drug development cycle cannot adapt to the rapid mutation of a single target, making it impossible to solve the problem of drug resistance (Alenazy, 2022). Therefore, in order to meet the clinical needs of anti-drug-resistant bacteria and anti-biofilms, it is important to develop a new generation of antibacterial agents that break away from traditional antibacterial mechanisms.

At present, small molecular drugs and traditional antibiotics are often unable to solve the clinical dilemma caused by multi-drug-resistant bacteria infections, so it is urgent to research and develop new types of antimicrobial agents. With the rapid development of technology, researchers have put forward many valuable concepts to build antibacterial and antibiofilm platforms, including cationic polymers, chitosan-based polymers, antimicrobial peptides, and nanomaterials such as metal/metal oxide-based nanoparticles (NPs). Different from the traditional small molecular antimicrobial agents, cationic polymers and nano-antimicrobial agents have become new antibacterial materials with excellent development potential because of their long active time, high chemical stability, and rare drug resistance. These new antibacterial materials work can be briefly classified into the following aspects. 1) loading small molecular antimicrobial agents, antimicrobial peptides, and other bactericidal ingredients; 2) showing inherent bactericidal activity by inflicting chemical or physical damage on bacteria, such as silver nanoparticles; 3) being activated by light to produce photothermal/photoacoustic and photodynamic effects; 4) being stimulated by peroxide or photoacoustic to produce a large number of reactive oxygen species (ROS); 5) combining two or more bactericidal modes to synergistically optimize the therapeutic effect.

In this review, we illustrate that how cationic polymers and nanomaterials could be used to combat bacterial infections. Firstly, the current main mechanisms of antibacterial activity of cationic agents and nanomaterials are comprehensively discussed. Secondly, the properties and design elements of cationic polymers and nanomaterials that exert good efficacy are reviewed, and the strategies of different antibacterial materials and the advantages and disadvantages of each antibacterial agent are presented (Table 1). Finally, the antibacterial materials displayed in this review were summarized and their clinical application prospects were discussed.

**TABLE 1:** Summary of mentioned antibacterial agents.

Category	Advantages	Disadvantages	Examples	Reference
Cationic polymers	Long active time; highly chemical stability	Poor penetration; rapid induction of host immunity; poor adhesion	Cationic polymers contained 31% ethyl methacrylate groups	Takahashi et al. (2017)
			Bile acid derivatives	Rahman et al. (2018); Rahman et al. (2020)
			Polymeric mimics of HDPs	Zhou et al. (2020)
			pH-responsive polymer-drug conjugate	Ye et al. (2020)
			Quaternary polyethyleneimine (QPEI) polymer	Hoque et al. (2019); Chen et al. (2021)
			Peptidomimetic polyurethanes	Vishwakarma et al. (2021)
			Polysaccharides-based polymer	Li et al. (2018a)
Chitosan-based polymers	Excellent adhesion; thermal stability; non-toxicity	Poor solubility under physiological pH	Chitosan-based hydrogel	Qu et al. (2018)
			Chitosan-based nanoparticle (NP) using quaternary ammonium chitosan	Hu et al. (2019)
			Cysteine-conjugated chitosan nanoparticles	Arif et al. (2018)
Antibacterial peptides	Natural positive charge; high antibacterial activity	High synthesis cost; high toxicity; poorly structural stability	AMPs conjugate with fluorophore groups	Li et al. (2017); Chen et al. (2018); Feng et al. (2015); Bao et al. (2021)
			AMPs loaded on hydrogels	Wei et al. (2021); Wu et al. (2022); Suo et al. (2021); Khan et al. (2019); Zhu et al. (2019); Moorcroft et al. (2020); Fjell et al. (2009); Wang et al. (2018); Cleophas et al. (2014a); Cleophas et al. (2014b); Liu et al. (2021)
			AMPs coated by nanomaterials	Sharma et al. (2018); Sharma et al. (2021); Salama. (2022); Pranantyo et al. (2021)
Metal/metal oxide-based NPs	High antibacterial activity; easy to modify and functionalization	Toxicity; easy to aggregate; poor stability	PEG- or PVP-coated AgNPs	Xiu et al. (2012)
			AgNPs combined with dialdehyde nano fibrillated cellulose	Li et al. (2019)
			Nano-Cu embedded in biodegradable polylactic acid polymers	Longano et al. (2012)
			Ag-Cu alloy nanomaterials	Jin et al. (2023)
			Surface-adaptive mixed charged zwitterionic 14 nm AuNPs	Hu et al. (2017)
			Chitosan coated zinc oxide nanocomposite containing rutin	Bharathi et al. (2019)
			ZnONPs covered titanium substrate	Wang et al. (2017)
Polymer-based NPs	Can be coated with other materials biocompatibility easy degradation		Vancomycin-loaded pH-responsive chitosan nanoparticles	Kalhapure et al. (2017)
			Naringin’s-cyclodextrin nanoparticles	Hussain et al. (2022)
			Oil-in-water cross-linked polymeric micelles	Landis et al. (2017)
			Polymer nanomicelles for modifying silver nanoparticles and curcumin coating	Huang et al. (2017)

## 2 Antibacterial mechanisms

The antibacterial mechanisms of cationic polymers and nano-antimicrobial agents are different from that of traditional antibiotics, so their damage to bacteria rarely leads to drug resistance. In this section, we briefly describe the antibacterial mechanisms of these two kinds of antimicrobial agents.

Currently, the majority of cationic antimicrobial polymers developed are amphiphilic macromolecules with surface active properties. Surfactants’ adsorption capacity, high binding affinity to bacterial cell membranes, and appropriate lipophilicity allow them to efficiently damage the structure of membranes and then cause cell lysis (Machado et al., 2017; Kohn et al., 2018; Saint Jean et al., 2018; Hitchner et al., 2021). For Gram-positive bacteria, the polymers cause the membrane’s destruction by diffusing inward through the cell wall and adsorbing onto the bacterial membrane. For Gram-negative bacteria, polymers are first adsorbed onto the outer membrane of the bacteria by electrostatic interactions and cause damage, which is reflected in the formation of voids and increased permeability. Second, the antimicrobial polymers spread through the cell wall and are adsorbed into the cell membrane, which eventually leads to the destruction of the cell membrane, resulting in the leakage of bacterial intracellular components and bacterial death. Poly-(α-aminoacids) (Engler et al., 2011; Hou et al., 2017), cationic polycarbonates (Yuen et al., 2017; Tan et al., 2020), phosphonium polymers (Loczenski Rose et al., 2017; Cuthbert et al., 2018; Palermo et al., 2019), chitosan-based cationic polysaccharides, quaternary ammonium salts (Li et al., 2021a), ε-poly-L-lysine (ε-PL) (Fan et al., 2023) are some examples of positively charged polymers with antibacterial properties.

**FIGURE 1:**
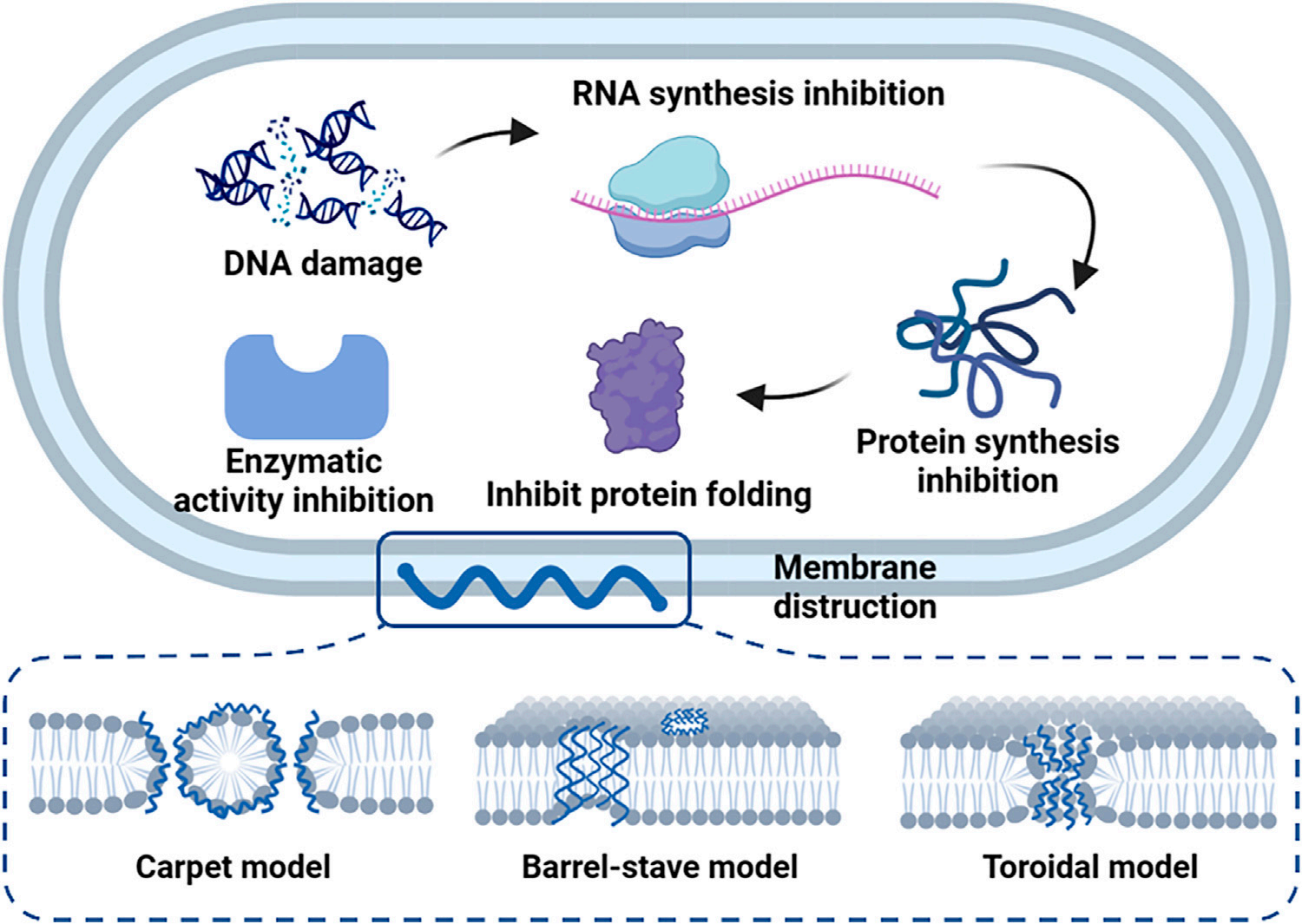
Antibacterial mechanisms of AMPs.

In particular, cationic antimicrobial polymers such as cationic antimicrobial peptides (AMPs) usually break bacterial cells through a series of steps, while some kinds of AMPs can enter the bacteria through cell penetration or endocytosis and interact with biological macromolecules to inhibit the growth of bacteria. AMPs can cause DNA damage, RNA synthesis inhibition, protein synthesis inhibition, protein folding inhibition, enzymatic activity inhibition, and cell wall synthesis inhibition (Figure 1). Several reviews have described in detail the mechanism of AMPs entering bacterial cells (Melo et al., 2009; Li et al., 2021b), including carpet models, barrel-stave model and toroidal model (Figure 1).

**FIGURE 2:**
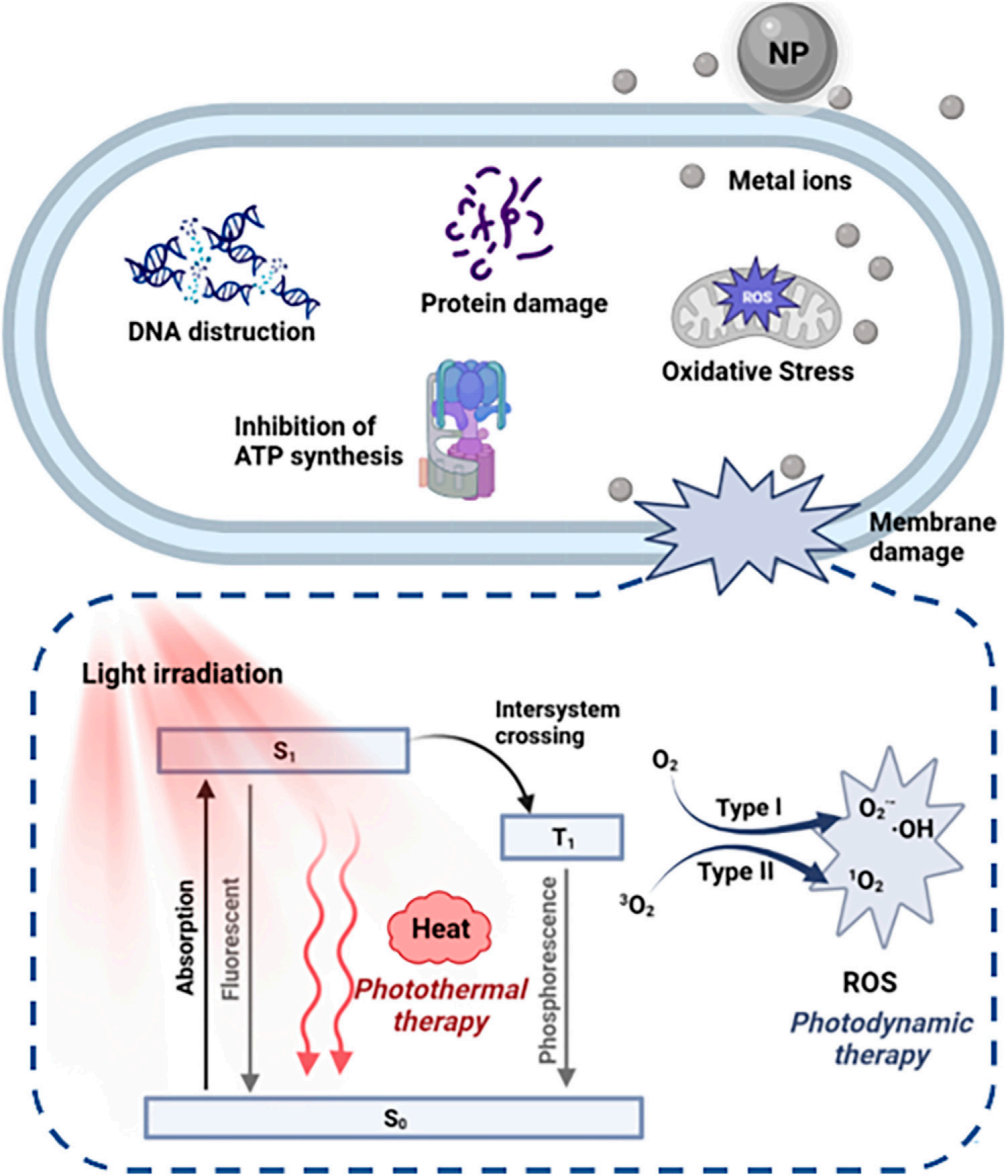
Antibacterial mechanism of metallic nanomaterials.

The ultra-small size and unique chemical properties of metallic nanomaterials provide advantages for the presentation of antibacterial activity. Metal nanomaterials have a variety of antibacterial mechanisms. Teichoic acid and lipopolysaccharide on the bacterial surface make them electronegative, which makes positively charged nanomaterials adsorb on the bacterial surface through electrostatic interaction (Gupta et al., 2019). As a result, cationic nanostructures have the opportunity to interact with the outer walls of bacteria and disrupt their structure. Compared to conventional antibiotics, cationic gold nanoparticles (AuNPs) bind more firmly to the surface of bacteria, which is the basis for their highly effective antibacterial activity (Xie et al., 2018; Li et al., 2020b). For some metal nanomaterials, especially silver nanoparticles (AgNPs), the antibacterial activity is mainly derived from the release of metal ions. The released metal ions not only contribute to the oxidative stress response in cells but also bind to other biomacromolecules in cells and cause dysfunction. Metal nanoparticles-mediated intracellular damage, including inhibition of ATP synthesis, depletion of reduced substances, decreased enzyme activity, and DNA destruction, also cause significant damage to bacteria (Figure 2) (Zhao et al., 2014; Buceta et al., 2015; Wang et al., 2021c). In addition, photodynamic therapy (PDT) and photothermal therapy (PTT) are two important antibacterial mechanisms of metal/metal oxide-based nanomaterials.

Photodynamic therapy (PDT) is a clinical procedure that uses reactive oxygen species (ROS) generated by photosensitizers to destroy surrounding biomolecules and kill the pathogenic bacterium (Jia et al., 2019; Liu et al., 2023). The manifestation of PDT is mainly mediated at the molecular level by two mechanisms: type I and type II (Figure 2). After photoactivation, the photosensitizer transitions from the ground state to the excited singlet state with a short lifetime, and then to the excited triplet state via a state-crossing process. Excited triplet photosensitive molecules react directly with surrounding substrates to produce free radicals or free radical ions, such as ·OH and O_2_^−^ through electron transfer (type I). In type I reactions of PDT, unsaturated phospholipid molecules in bacterial cell membranes are predominantly hydrogenated. Further chemical reactions between molecules and oxygen result in lipid peroxides, which compromise the structural integrity of bacterial cell membranes and raise membrane permeability (Silva et al., 2018). To create ^1^O_2_, excited triplet PSs molecules can also exchange energy with molecules of oxygen. As ^1^O_2_ is the most active ROS species, it directly damages biological molecules like proteins, enzymes, DNA, and other cellular components through oxidative stress, essentially killing bacteria (Type II) (Wang et al., 2019). Therefore, compared with other therapies without photoactivation, PDT shows the following advantages: 1) more targeted treatment through the application of light; 2) not limited by the concentration of H_2_O_2_ in the microenvironment of the infected site; 3) light-induced ^1^O_2_ is more threatening than other ROS (·OH and O_2_^−^), making it more effective in antibacterial effect.

Under light excitation, photosensitizers can be used as photodynamic antibacterial materials to kill bacterial cells through light-dependent ROS production. In addition, some materials can decay back to the ground state by emitting fluorescence, or produce thermal energy by consuming energy through non-radiative relaxation pathways, namely the photothermal effect (Figure 2). These processes may be single molecules, or they may be caused by the collision of excited singlet particles with their surroundings. Ideally, excellent photothermal materials should be able to meet three key requirements: 1) strong absorption efficiency in the near-infrared region (to promote effective light absorption in the highly penetrating spectral region of the tissue), while low fluorescence quantum yield and low reactive oxygen generation efficiency. 2) Non-toxic under dark conditions, and produces a large amount of heat energy under near-infrared light. 3) Can be quickly metabolized by the body without producing toxic metabolites. Apart from metal/metal oxide-based nanoparticles, many organic dyes, including cyanines and carbon nanotubes, as well as some polymers, have been found to meet these requirements and have been studied as potential PTT agents (Jung et al., 2018).

In addition to their direct use as antimicrobials, metallic nanomaterials have also been used as nanocarriers to transport existing antimicrobials. Antimicrobial agents can be loaded onto metal nanoparticles’ surfaces by covalent attachment or self-assembly (Hu et al., 2018; Simon-Yarza et al., 2018). In summary, as novel nano-antibacterial materials, metallic nanoparticles offer a variety of antimicrobial pathways to fight against superbugs and circumvent mechanisms of drug resistance.

## 3 Bioactive agents for combating bacterial infections

### 3.1 Polymer-based agents for combating bacterial infections

#### 3.1.1 Cationic organic antibacterial polymers

Cationic agents are a group of therapeutic agents carrying positive charges which can be naturally derived or synthesized. The positively charged agents are shown to interact with negatively charged bacterial membranes and can be imported into bacterial cytoplasm (Severina et al., 2001). For many antibacterial agents, the formation of biofilm greatly limits their penetration and effective accumulation to the bacterial cells and greatly dampens their performances (Koo et al., 2017). Therefore, cationic agents can be rationally constructed to foster their penetration into bacterial biofilm colonies and achieve targeted drug delivery or local release of bactericidal agents. Moreover, owing to their well-defined structures, promising biocompatibility, accessibility, and ease of production, the research on cationic organic agents like cationic polymers, hydrogels, chitosan, and antibacterial peptides has attracted great attention in the development of novel strategies to counter bacterial and their biofilm-associated infections.

Cationic polymers are a group of supramolecular systems carrying a significant amount of positively charged sites on the surface and can therefore interact with the negatively charged bacterial cells. Their efficacy against planktonic bacterial species has been widely explored. In particular, gram-negative pathogens are known to be a major health burden, especially with the rapid emergence of antimicrobial-resistant strains. For example, many studies constructed amphiphilic polymer systems containing a significant portion of cationic residues and therefore exhibit significant electrostatic interaction with the anionic bacterial membrane. Nguyen et al. (2017) reported self-assembled single-chain polymeric nanoparticles (SCNPs) via RAFT polymerization of amphiphilic ternary copolymers that comprised oligo ethylene glycol, amine groups, and hydrophobic residues. The copolymer exhibited excellent antimicrobial efficacy, eradicating >99% planktonic and biofilm-associated bacterial cells within an hour at micromolar level concentrations owing to the synergistic action of the aforementioned three functionalities. Likewise, using RAFT polymerization, Takahashi et al. (2017) synthesized cationic polymer PE_0_ and copolymer PE_31_ that contained 31% ethyl methacrylate groups. Both polymers displayed good antibacterial activity against *S. mutans* and effectively killed the planktonic bacteria. At a high enough concentration (1,000 μg/mL), both polymers were able to significantly diminish biofilm biomass by 80% upon two-hour incubation.

**FIGURE 3:**
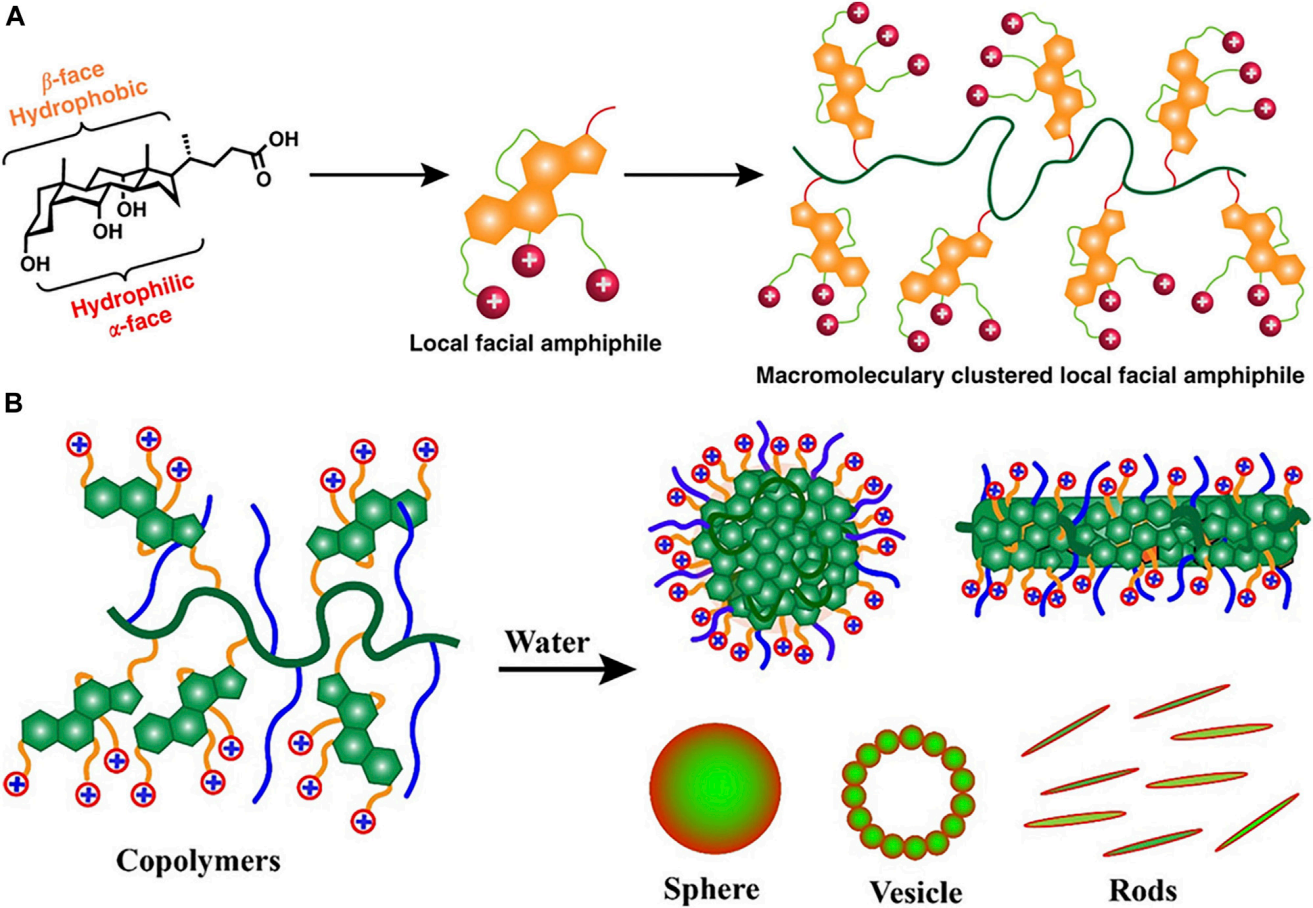
**(A)** The amphiphilic cationic polymers composed of cholic acid as repeat units. Reproduced from (Rahman et al., 2018) with permission. **(B)** Amphiphilic copolymers of bile acids and polyethylene glycol formed nano-aggregates in water. Reproduced from (Rahman et al., 2020) with permission.

To mimic naturally occurring host defense peptide (HDP) that mediates selective membrane disruption of negatively charged bacterial membrane over zwitterionic mammalian cell membranes, a synthetic polymer with cationic charges is developed. However, many studies utilize AMP-mimicking polymers based on a global amphiphilic framework that cannot be fine-tuned for the selective killing of bacterial cells. To address this, Rahman et al. (2018) constructed multiple bile acid derivatives-cholic acid, lithocholic and deoxycholic-that carries variable ammonium charges as shown in Figure 3A. Recently, the authors further explored the effect of altering polymer architecture on the selectivity of cationic polymer agents (Figure 3B) (Rahman et al., 2020). The hydrophobicity and hydrophilicity are balanced by introducing cholic acid-carrying ammonium charges. The self-assembled polymer showed broad-spectrum antibacterial activity and low hemolysis activity against the mammalian cell. These studies elucidate the potential of cationic polymer agents in the treatment of drug-resistant bacterial infections.

To ensure the polymeric mimics of HDPs can be used therapeutically, it is vital to develop such polymers with low *in vivo* toxicity and high biocompatibility. One such polymer is poly (2-oxazoline) (POX) which has been explored and shown to be non-toxic and biocompatible. Zhou et al. (2020) used a carbonyl translocation strategy to design and synthesize a peptidomimetic cationic polymer Gly-POX via cationic ring-opening polymerization (CROP). The polymer utilized the biocompatibility of the POX backbone and enhanced the bioavailability and stability of HDPs owing to POX’s resistance to enzymatic degradation and proteolysis. The polymer demonstrated potent *in vitro* and *in vivo* antibacterial efficacy against *MRSA*-biofilm infection.

**FIGURE 4:**
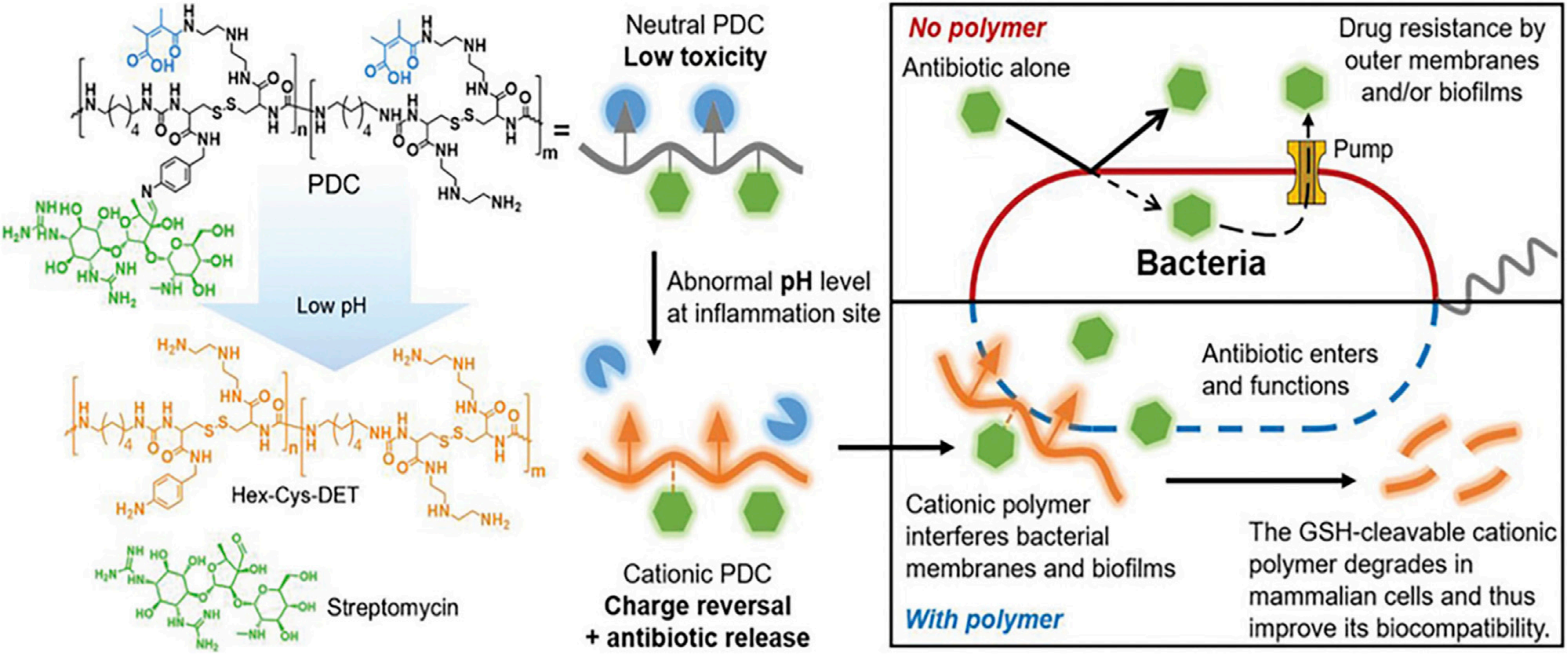
Schematic representation of the antibacterial mechanism of PDC. Reproduced from (Ye et al., 2020) with permission.

Typical cationic polymers might induce broad non-specific toxicity due to electrostatic interactions of the positively charged polymers with biomacromolecules, membranes, and extracellular materials. Therefore, responsive charged polymers that can be rationally configured with “on-demand” antimicrobial and antibiofilm abilities are becoming a promising strategy. Chronic biofilm infections are often associated with inflammation and local bacterial fermentation that would induce an acidic microenvironment that can be exploited by pH-sensitive polymers. Ye et al. (2020) reported a pH-responsive polymer-drug conjugate for antimicrobial therapy. Streptomycin was linked to the cationic polyurea polymer Hex-Cys-DET via a pH-responsive imine bond which is responsible for the charge reversal at the site of infection to release the functional cationic polymer as shown in Figure 4. The polymer was reported to have low MIC against multiple pathogenic biofilm-forming bacteria strains with low hemolytic activities.

**FIGURE 5:**
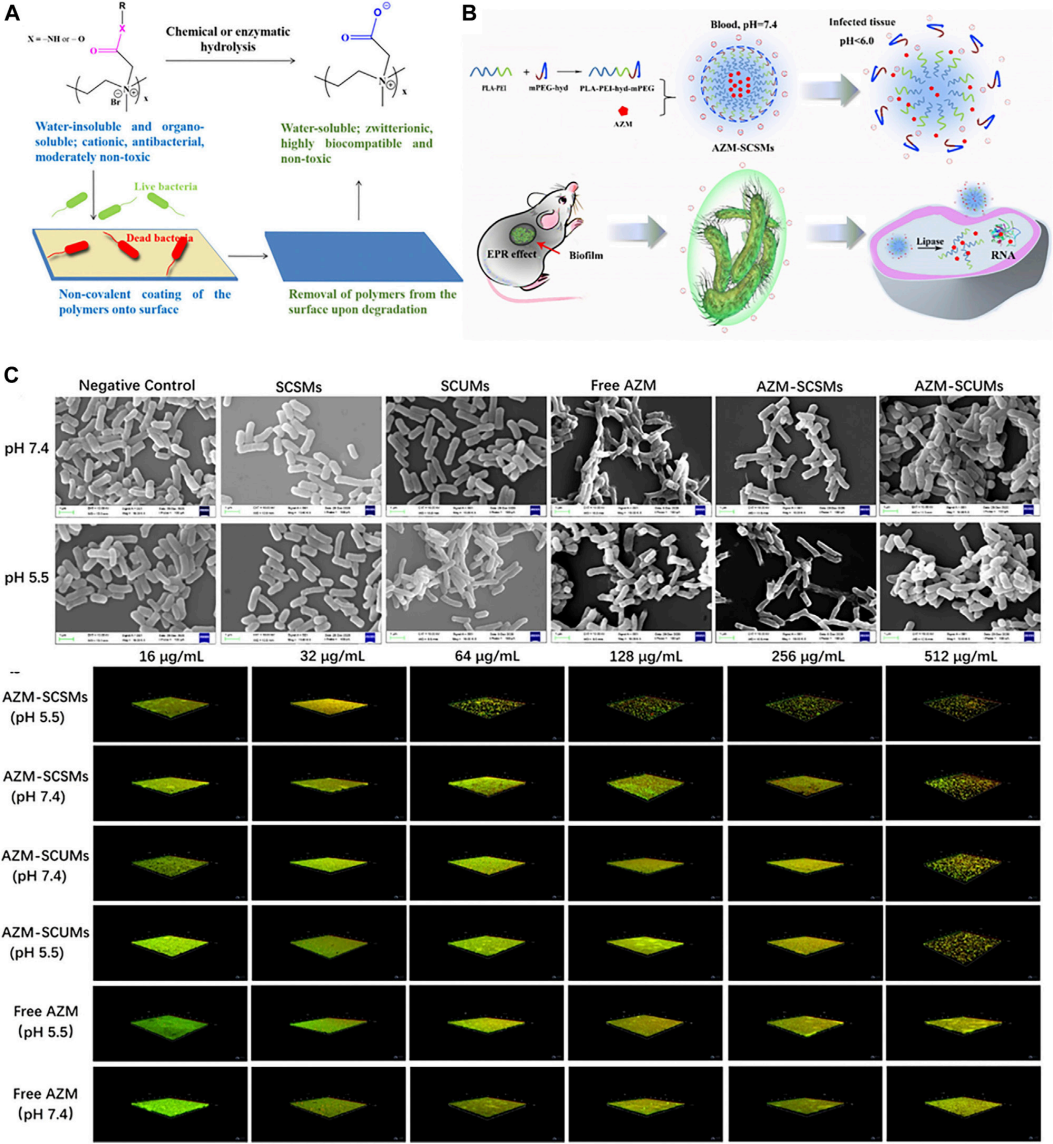
**(A)** Schematic illustration of the charge-switchable antibacterial paint. Reproduced from (Hoque et al., 2019) with permission. **(B)** Schematic representation of the structure of AZM-SCSMs and the mechanism for the treatment of biofilm-associated infection. **(C)** SEM micrographs of *P. aeruginosa* and CLSM imaging of *P. aeruginosa* biofilm with different treatments. Reproduced from (Chen et al., 2021) with permission.

One of the most significant healthcare burdens of bacterial infection is the development of biofilms on medical devices and implants. Hoque et al. (2019) developed a quaternary polyethyleneimine (QPEI) polymer whose active cationic polymer form can be rendered zwitterionic polymer under acidic conditions (Figure 5A). With both forms demonstrating the compelling antibacterial activity, the hydrolyzed product of QPEI showed no *in vitro* or *in vivo* toxicity against mammalian cells. Though demonstrating the potential to counter biofilm-associated biofilm infection, many polymeric micelles face the challenge of rapid host immune response and degradation that leads to low bioavailability and short blood circulation time. Similar strategies can be applied to construct on-demand micelles that target specific biofilm life cycles. Chen et al. (2021) reported a polymeric micelle constructed by PLA-PEI-hyd-mPEG linked with an arylhydrazone bond that can reduce mononuclear clearance and preferentially switch to cationic forms in low-pH infection sites (Figure 5B). The micelles were also loaded with azithromycin and demonstrated good biocompatibility and antibiofilm potency. The charge-switchable polymer demonstrated effective biofilm penetration with antibiofilm efficacy confirmed both *in vitro* and *in vivo* and could be applicable as an advanced drug delivery strategy against biofilm infections (Figure 5C).

Although various strategies have been explored to prevent or suppress the formation of biofilms, it remains a major challenge to remove surface-established biofilms. Current treatment relies on mechanical and physical disruption, which has the potential to further spread and exacerbate the infection to other surfaces (Koo et al., 2017). On the other hand, Vishwakarma et al. (2021) constructed peptidomimetic polyurethanes to augment bacterial surface motility, prevent surface attachment and disrupt established biofilms of various clinically relevant strains like *MRSA* at subinhibitory concentrations.

Li et al. developed a biofilm-dispersing cationic polymer using polysaccharides. The copolymer effectively ameliorated biofilm infection of various pathogenic like *MRSA* and vancomycin-resistant *Enterococci* species and has superior potency compared to conventional antimicrobial agents (Li et al., 2018a). The assembly of DA95B5 polymer into nanoparticles enabled deep biofilm penetration and subsequent bacterial detachment, thereby eliciting its dispersal effect. Nevertheless, the wider assumption regarding biofilm dispersal remains that dispersed bacterial cells return to a phenotype similar to that of active, planktonic cells, making them susceptible to conventional antimicrobial agents and antibiotics (Koo et al., 2017). Hence, this copolymer could still be used in conjugate with other agents that are effective in killing planktonic bacterial species.

For a long time, the research of cationic polymers has extended from natural polymers to synthetic polymers, which usually have the advantages of long active time and high chemical stability. However, most cationic polymers still have problems such as poor penetration and rapid induction of host immunity, and further research is needed to improve antibacterial activity and biocompatibility. Furthermore, cationic polymers are also being conjugated with nanoparticles to disrupt preformed bacteria biofilm and enable the effective killing of biofilm-associated bacteria cells, and will be covered in subsequent chapters.

#### 3.1.2 Chitosan-based polymers

Although cationic polymers like polyethyleneimine (PEI) have demonstrated promising *in vitro* efficacy against biofilm infections, the *in vivo* efficacy of such polymers might be limited by the rapid induction of host immune responses by polycationic agents which reduces blood circulation and limit the effective concentrations at the site of biofilm infection. Therefore, chitosan-based polymer systems have attracted great attention with promising efficacy against biofilm infections and adhesive properties.

A major drug administration hurdle in wound biofilm treatment is poor infection site adhesion and limited biofilm penetration. Therefore, polymers need to be designed to exhibit adhesive properties besides good antibiofilm efficacy. Qu et al. (2018) tackled this challenge by constructing adhesive curcumin-loaded hydrogel with antimicrobial effects. The polymer achieved promising stretchability (58.2%–76.1%) comparable to native skin. The superior therapeutic efficacy against biofilm colonies was verified in *in vivo* models, showing great promise as a chitosan-based wound healing material in biofilm-related infections.

In oral biofilm-associated infections lead to an acidic microenvironment. Exploiting this characteristic, Hu et al. synthesized a novel chitosan-based nanoparticle (NP) using quaternary ammonium chitosan, N, N, N-trimethyl chitosan (TMC) along with liposome and doxycycline, namely TMC-Lip-DOX NPs (Hu et al., 2019). The NPs showed potent interaction with oral biofilm colonies and effective membrane destruction in *in vivo* mouse models while having good biocompatibility in mammalian cell lines. Another study investigated mouthwashes incorporated with chitosan content in alleviating biofilm formations of oral microorganisms (Costa et al., 2015). Chitosan mouthwash was effective in suppressing microbial adherence, biofilm formation, and disrupting established biofilms of *Streptococcus mutans*. However, the study was restricted to *in vitro* evaluations.

Although pH is commonly used for the targeted delivery of therapeutic agents, under acidic conditions, many polymers suffer from poor retention time due to degradation and inactivation. Chitosan-based materials with excellent biocompatibility, mucoadhesive properties, and feasibility for further chemical modifications make them ideal for oral drug delivery to GIT. Arif et al. (2018) reported cysteine-conjugated chitosan nanoparticles amoxicillin-Cys-CS/PMLA for targeted delivery to eradicate *H. pylori*. The pH-sensitive properties of the polymer allowed the postponed release of amoxicillin to enhance its local concentration at infection sites of GIT. The nanoparticle demonstrated effective inhibition of *H. pylori* growth while exhibiting minimal toxicity against mammalian cells.

In summary, owing to its advantageous properties like stretchability, biocompatibility, high density of functional groups, mucoadhesive, and degradability, chitosan and its derivatives are shown to be a promising antibiofilm strategy. Its wider clinical translation as combination therapy could provide further insights into its efficacy in the eradication of established biofilms and associated chronic infections.

### 3.2 Antimicrobial peptides for combating bacterial infections

Antimicrobial peptides (AMPs) are small molecular proteins with antimicrobial activity in organisms in nature, which are an important line of defense against pathogens. The main pathways of their action are bacterial cell membrane destruction, and immune and inflammatory response regulation (Mookherjee and Hancock, 2007). Recently, an increasing number of AMPs have entered clinical trials (Koo and Seo, 2019), and have shown the promising role of AMPs in therapeutic applications. Here, we review different applications of AMPs in the study of antimicrobial activity.

**FIGURE 6:**
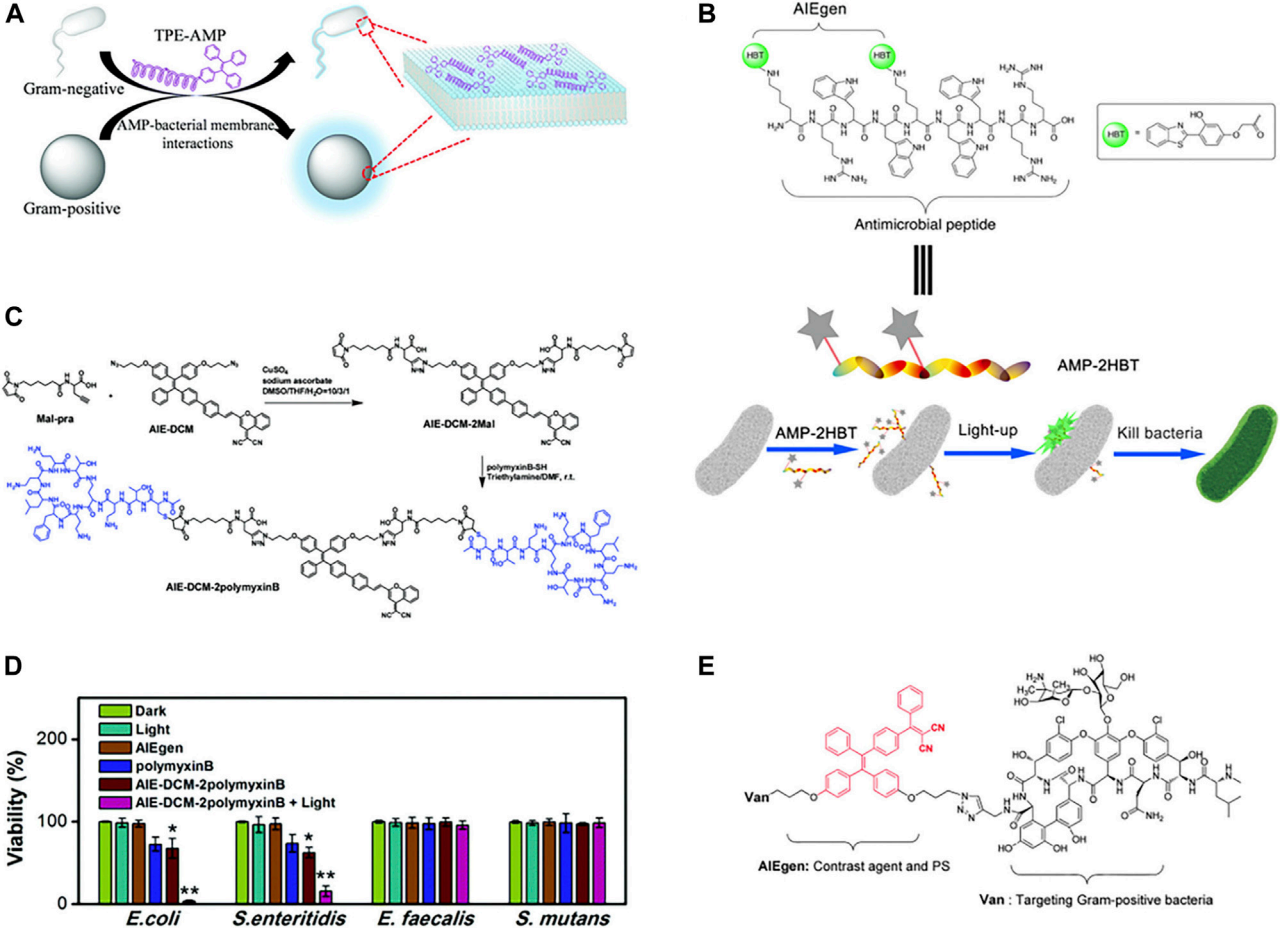
**(A)** Summary of the interaction between TPE-AMP with bacterial membranes. Reproduced from (Li et al., 2017) with permission. **(B)** Schematic illustration of the bacterial imaging and killing mechanism of AMP-2HBT. Reproduced from (Chen et al., 2018) with permission. **(C)** Synthetic route to AIE-DCM-2polymyxinB. **(D)** Quantitative biocidal activities of various treatments towards *E. coli*, *S. enteritidis*, *E. faecalis,* and *S. mutans*. Reproduced from (Feng et al., 2015) with permission. **(E)** Chemical structure of AIE-2Van. Reproduced from (Bao et al., 2021) with permission.

To reveal the bactericidal mechanism of AMPs, monitoring the dynamic interaction between AMPs and bacteria in real-time is necessary. Due to its high sensitivity and easy operation, fluorescence imaging technology has been widely used to visualize the dynamic interaction between molecules and organisms in the microscopic environment (Bunschoten et al., 2013). Linking the AMP (CysHHC10) to the AIE fluorophore group (TPE), the fluorescence emitted by TPE induced by the surface enrichment of these peptides due to the interaction between AMPs and the bacterial membrane could be used to visualize the interaction between bacteria and AMP (Figure 6A) (Li et al., 2017). However, the introduction of fluorescent groups altered the physicochemical properties of AMPs, making them less effective against bacteria. Different from modification with fluorescent groups at the end of antimicrobial peptides. Chen et al. modified HBT on the amino group of the side chain of HHC36 (Figure 6B), showing good luminescent fluorescence after binding with bacteria, which could monitor the binding process in real-time and has good antibacterial activity (Chen et al., 2018). This AIE active probe provided a convenient tool to study the bactericidal mechanism of AMP. Furthermore, some antibiotics that has been used in clinical practice, such as peptide antibiotic polymyxin B and glycopeptide antibiotic vancomycin, had also been used in AIE fluorophore modification to construct new luminescent probes that could recognize and kill bacteria (Figures 6C–E) (Feng et al., 2015; Bao et al., 2021). Selective identification of bacteria using vancomycin and polymyxin B targets on bacteria, detection of the bacteria using the luminescence properties of the AIE group, and enhancement of antibacterial activity through the photodynamic sterilization properties of the AIE group.

**FIGURE 7:**
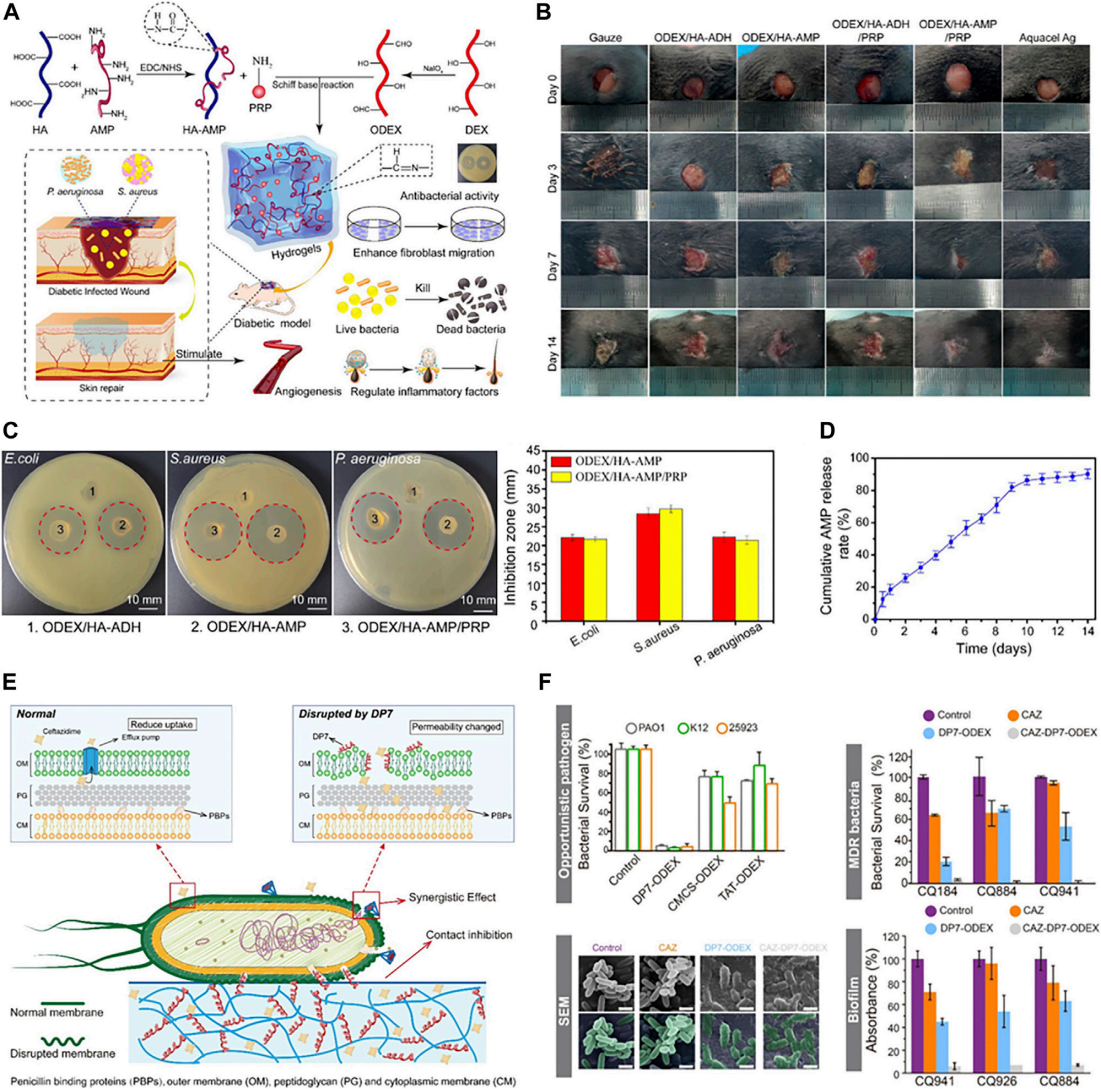
**(A)** Schematic illustration of the application of ODEX/HA-AMP hydrogel in the treatment and healing promotion of infected wounds. **(B)** Photographs of wounds with different treatments on days 0, 3, 7, and 14. **(C)** Typical inhibition zones of hydrogels against *E. coli*, *S. aureus,* and *P. aeruginosa*. **(D)** The cumulative release curve of AMP from ODEX/HA-AMP hydrogel. Reproduced from (Wei et al., 2021) with permission. **(E)** Schematic representation of antibacterial mechanism. **(F)** *In vitro* antibacterial properties. Reproduced from (Wu et al., 2022) with permission.

Most AMPs have amino groups that readily form amine bonds with carbonyl groups, and hydrogels are often cross-linked based on imine bonds. Therefore, using hydrogel as an application form of AMPs is a promising strategy for antibacterial infection. It is very common in hydrogels with antimicrobial peptides as skeletons. Wei et al. (2021) modified Cecropin on hyaluronic acid with an imine bond and then mixed it with platelet-rich plasma (PRP) and oxidized dextran (ODEX) under physiological conditions to prepare hydrogels (Figure 7A). The hydrogel shows good antibacterial activity *in vivo* and *in vitro*, and also has the function of promoting wound healing (Figures 7B–D). Similarly, due to the abundance of amino groups, the AMP DP7 might form hydrogels by forming imine bonds with the carbonyl group in ODEX, while loading ceftazidime into the hydrogels. The disruption of the outer membrane of *P. aeruginosa* caused by DP7 allowed ceftazidime to cross the outer membrane barrier of gram-negative bacteria. As a result, ceftazidime was able to bind to the penicillin-binding protein on the inner membrane and act in a synergistic antibacterial effect with DP7. In addition, the combination of DP7 gave ceftazidime the ability to resist *P. aeruginosa* biofilms (Figures 7E, F) (Wu et al., 2022).

The bacterial infection wound has a slightly acidic environment. The AMP [KK(SLKL)_3_KK] was cross-linked with oxidized hyaluronic acid via Schiff base to form a hydrogel and was released in the slightly acidic microenvironment of the bacterial infected wound. The composite hydrogel was injectable, had high biostability and strong mechanical strength, showed excellent *in vitro* and *in vivo* broad-spectrum antibacterial activity, and promoted wound healing properties, providing an effective strategy for the treatment of chronic bacterial infections in wounds (Suo et al., 2021). Khan et al. prepared hydrogels based on AMPs [epsilon-poly-L-lysine (EPL)] and catechol. Results from the treatment of *MRAB* infection in a mouse model of a burn wound on the back skin showed that hydrogel had a clear scavenging effect on the wound bacteria and was expected to be a biological agent against multi-drug resistant bacterial infections (Khan et al., 2019). Compared to the AMPs in the hydrogel skeleton, the AMPs loaded on the surface of the hydrogel can reach the infected wound more effectively, which improves the utilization efficiency of the AMPs. The cationic short peptide p3 (RRRFRGDK) with antibacterial activity was directly connected to the surface of the hydrogel through an amidation reaction, which enhanced the antibacterial activity and hemostatic ability without affecting the appearance or internal structure of the original hydrogel, and showed good wound healing promotion performance in the treatment of infected wound model in mice (Zhu et al., 2019). To prevent the antimicrobial peptides from degrading by protease, Moorcroft et al. coated the antimicrobial peptide IK8 (IRIKIRIK-CONH_2_) in some liposomes and loaded it into a hydrogel of PEG and gold nanorods. The 860 nm laser stimulated the gold nanorods to lyse the liposomes through the photothermal effect, thereby releasing antimicrobial peptides and producing bactericidal activity against *P. aeruginosa* (gram-negative bacteria) and *S. aureus* (gram-positive bacteria) (Moorcroft et al., 2020).

Infection of hospital medical equipment is common, so the demand for safe and non-toxic medical device coatings has increased significantly. HHC10 is an antimicrobial peptide screened by computer-aided drug design with good antibacterial activity (Fjell et al., 2009). A hydrogel based on sodium alginate nuclear polyethylene glycol, and the AMP (CysHHC10) was grafted onto the hydrogel by thiol-ene reaction, which had satisfactory antibacterial properties and good biocompatibility (Wang et al., 2018). Cleophas et al. used the thiol-ene click reaction to load CysHHC10t in the polyethylene terephthalate surface hydrogel network. It has good bactericidal activity against *S. aureus* and *S. epidermidis* which are often associated with infection of biological materials (Cleophas et al., 2014a). In addition, this work team covalently attached HHC10 to a PEG-based hydrogel coating that was capable of killing *S. aureus*, *S. epidermidis*, and *E. coli* with low hemolysis (Cleophas et al., 2014b). Liu et al. (2021) coated antibacterial peptide WR (WRWRWR-NH_2_) or Bac2A (RLARIVVIRVAR-NH_2_) hydrogels on clinical medical devices directly in contact with blood to provide good bactericidal activity and prevent bacterial adhesion. The *in vitro* and *in vivo* evaluation of the anti-infective showed promising antibacterial effects.

Free peptides are easily degraded by proteases present in organisms and are more toxic when used alone. For example, Sharma et al. (2018) encapsulated HHC10 in nanoparticles and studied the antibacterial effect and possible mechanism of HHC10 on *E. coli*. The results showed that HHC10-NP could be bactericidal through the multi-mode mechanism, that was, it could directly interact with bacteria to dissolve its membrane and cause its death by inducing apoptosis pathways in host cells. In addition, HHC10-NP was significantly less cytotoxic to RAW264.6 than nude HHC10. Inspired by this result, they encapsulated HHC-8 (KIWWWWRKR) and MM-10 (MLLKKLLKKM) in polycaprolactone nanoparticles and investigated the effect of the nanoparticles on the activity of the AMPs. The results showed that the protection provided by AMP encapsulation increased the membrane permeability of antimicrobial peptides and enhanced the accumulation of antibiotics in *mycobacterium*, thus producing a synergistic effect (Sharma et al., 2021). Salama (2022) coated chitosan nanoparticles (CS-NPs) with a novel potent ultra-short antimicrobial peptide. ​The antibacterial activity of WRWRWR-CS-NPs has been shown to be highly selective and durable against gram-positive bacteria, including clinically resistant strains, with little toxicity to normal cells. Since bacterial membranes are more negatively charged than eukaryotic membranes, nanosystems that respond to bacterial membranes but do not respond to eukaryotic membranes can be designed according to the differences in membrane charges. Pranantyo et al. (2021) neutralized the strong positive charge of antibacterial peptide-modified gold nanoparticles by polyanionic poly (p-phenylene ethynylene) (PPE), which was not toxic to normal cells with a low negative charge. However, bacteria with strong negative charge could competitively replace PPE, allowing the antibacterial peptides and gold nanoparticles inside to play a powerful antibacterial role.

Most antimicrobial peptides achieve broad-spectrum antibacterial activity by depolarizing and penetrating bacterial cell membranes, and their activity can be enhanced by coupling with cell-penetrating peptides (CPPs). Compared with antimicrobial peptide magainin, CPP (R9)-magainin conjugate effectively crosses the lipid bilayer, increasing antibacterial activity by 2–4 times against gram-positive bacteria and 4–16 times against gram-negative bacteria (Lee et al., 2019a). In addition, CPPs could contribute to the use of antimicrobial agents in intracellular bacterial therapy (Li et al., 2018b). Many CPPs are similar to AMPs and have amphiphilic and cationic structural characteristics. Among them, cyclic peptide [R_4_W_4_] had strong antibacterial activity against *MRSA* and *P. aeruginosa*. At the same time, when combined with tetracycline, it could enhance the antibacterial effect of tetracycline and had a significant effect on a variety of drug-resistant bacteria (Oh et al., 2014).

Many antimicrobial peptides are host defense peptides from natural plants and animals, but many of them can be optimized for optimal antimicrobial effects. A large number of peptides were screened by the high throughput method. Based on the linearized variant of bovine bactenecin (RLARIVVIRVAR-NH_2_), a complete replacement library of 12 amino acid peptides was screened and optimized 12-Mer peptides were designed by substituting each amino acid with the most favorable substitution. It had excellent antibacterial activity against *E. coli* (Hilpert et al., 2005). Design and synthesis of antimicrobial peptides based on natural product scaffolds is a common approach for antimicrobial peptide development. Luther et al. designed an antibiotic containing a polymyxin-similar structure with antibacterial activity against a variety of gram-negative bacteria, which was expected to overcome the resistance of colistin (Luther et al., 2019).

Hancock’s group successfully performed computer simulation screening of antimicrobial peptides using a combination of quantitative structure-activity relationship (QSAR) and machine learning techniques. Antimicrobial peptides, including HHC10 and HHC36, with comparable or even higher activity than many traditional antibiotics, were obtained (Cherkasov et al., 2009; Fjell et al., 2009). They investigated the potential of HHC10 to reduce periapical inflammation as a possible adjunct to pulp therapy (Lima et al., 2021). In later studies, Llamas-Gonzalez et al. (2013) explored HHC10 antibacterial activity of *Mycobacterium tuberculosis*. The results of *in vivo* and *in vitro* activity showed that HHC10 could be used for anti-tuberculosis drugs. To understand the mechanism underlying the biological activity of HHC-36, Nichols et al. (2013) investigated the conformational properties of the peptide and its interaction with bacterial membranes or mammalian cell membranes. The results suggested that Trp residues play an important role in the formation of the amphiphilic structure of antimicrobial peptides. The peptide was first attached to the membrane surface by electrostatic attraction, and then the Trp residue was inserted into the internal region.

The prevention of bacterial infection in joint implants is a major challenge in joint replacement, and the use of antibacterial coatings loaded with antimicrobial peptides in implants is a promising strategy. Hancock and his team studied *in vitro* drug release, antibacterial performance and cytotoxicity, and *in vivo* bone growth of an AMP loaded with a calcium phosphate-coated titanium (CaP-AMP) implant in a rabbit model. The results showed rapid release of antimicrobial peptides in the body for the first few hours, followed by slow release. It had good antibacterial activity against *S. aureus* and *P. aeruginosa* but had no effect on bone growth on the implant (Kazemzadeh-Narbat et al., 2012). The team then refined the implant by impregnating thin layers of titanium dioxide NT and CaP coatings with HHC36. These films were covered with a thin phospholipid (POPC) film to control AMP release from the bioexcited film, thus making AMP release more controlled and sustained. It also prevents bacteria from forming biofilms on the surface of the implant (Kazemzadeh-Narbat et al., 2013). In addition, Abbasizadeh et al. (2020) attached HHC36 to a nanofiber hybrid composite consisting of silk fibroin (SF)/hydroxyapatite (HA) directly onto a titanium plate via electrospinning. The device was non-toxic to MG-63 cells and maintain antibacterial activity for 21 days. To solve the problem of bacterial infection in transdermal implants, Miao et al. (2021) constructed HHC-36 sustained-release system coated with poly D, L-lactic acid (PDLLA) and polylactic acid glycolic acid (PLGA). It could maintain effective release *in vitro* for 15 days and is effective against biofilms.

Cationic antimicrobial peptides have a natural positive charge, which is the key to their strong antibacterial activity. However, this strong positive charge is also a source of its biological toxicity, and the introduction of guanidine groups into its structure may be helpful in balancing its antibacterial activity and biological safety. Unfortunately, the high synthesis cost and unstable chemical structure also limit the widespread use of cationic antibacterial peptides.

### 3.3 Nanomaterials for combating bacterial infections

Nanotechnology has become one of the most rapidly growing areas of science and technology in the whole world. Nanomaterials are organic, inorganic or hybrid particles with dimensions in the 1 nm and 100 nm range (Vert et al., 2012). The emergence of nanomaterials has brought many changes to the development of technology and industry. Their high surface area provides the premise for their new properties, including magnetic, electro-optical, and so on (Whitesides, 2005). In fact, nanotechnology has found its way into treatment options for many diseases, and its involvement in drug development has led to the development of a class of materials called “nanomedicine”.

Nanomaterials are small enough that they can penetrate the membrane structure to reach the interior of bacteria, causing damage to biomacromolecules such as nucleic acids and proteins in their cells, and can also penetrate the biofilm for ablation. Up to now, a large wide range of nanomaterials have been applied to combat bacterial infections in order to get around the limitations of traditional antibiotic therapy, such as metal or metal oxide-based nanoparticles, polymers, liposome, carbon-based nanoparticles, nanoemulsions, microneedles and so on (Ageitos et al., 2017; Yuan et al., 2018). In this chapter, we will summarize some recent developments of nanomaterials used in this field.

#### 3.3.1 Metal/metal oxide-based nanoparticles

Among these nanomaterials, nanoparticles based on metals or metal oxides have attracted considerable attention in the field of antibacterial. The inherent advantages of metals and metal oxide-based nanoparticles, including their small size, controllable morphology, and easy surface modification, all help them become good antibacterial materials. At the same time, it also has unique optical, chemical, and physical properties. Their use in antibacterial activity has been extensively investigated during the past 20 years as nanoscience has advanced. A variety of metals are known to have intrinsic antimicrobial properties and are not prone to resistance. Furthermore, their corresponding metal/metal oxide nanoparticles have stronger antibacterial activity (Hajipour et al., 2012). The general antibacterial mechanisms of these metal nanoparticles mainly include disrupting cell membrane metabolism, penetrating cell membranes, and disrupting enzymatic activity, generating (ROS), or attaching to cell membranes to reduce bacterial replication. The detailed antimicrobial mechanisms have been elaborated on in the second part of the content.

**FIGURE 8:**
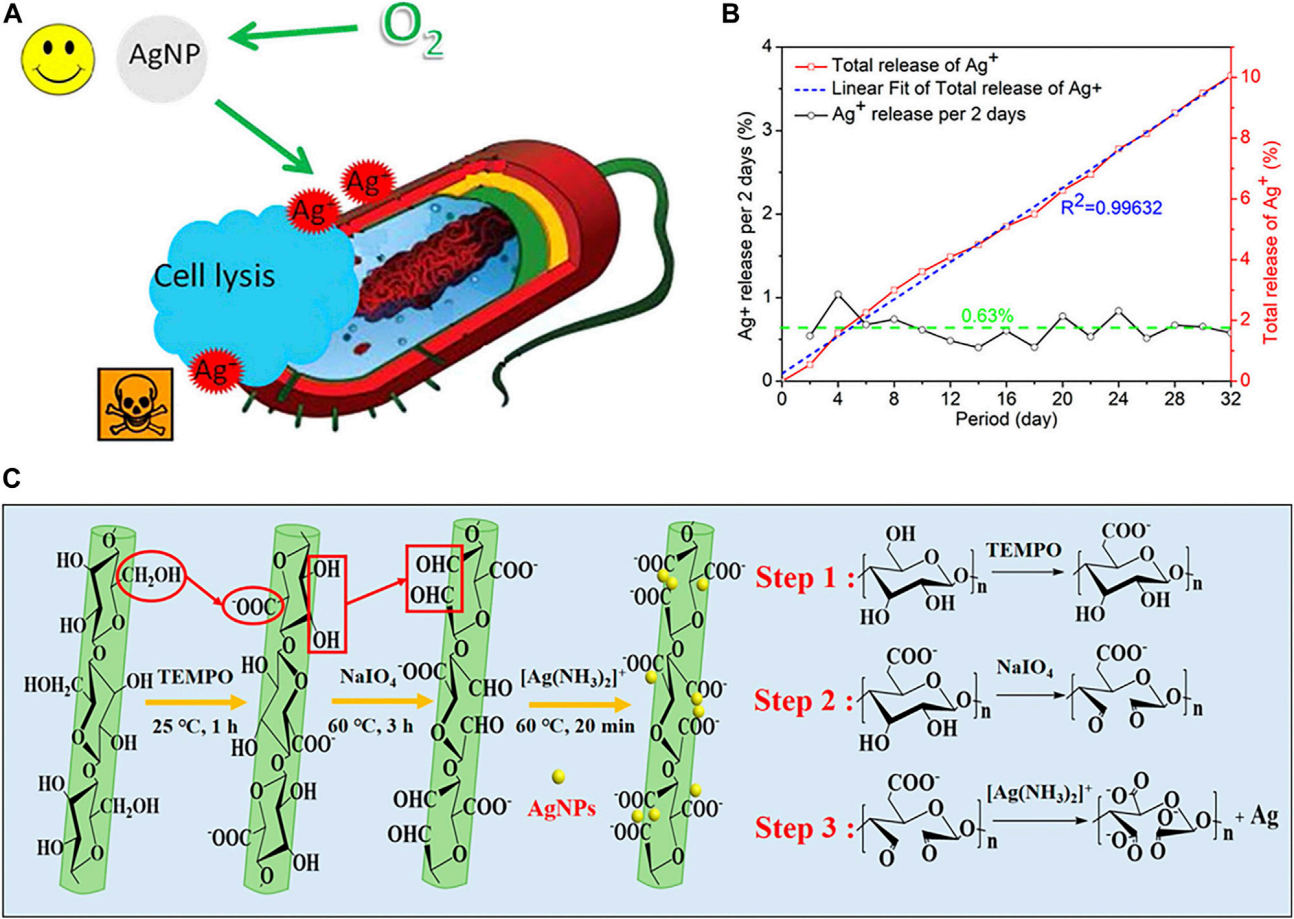
**(A)** Schematic of AgNPs, Ag^+^, and cell interaction. Reproduced from (Xiu et al., 2012) with permission. **(B)** The release rate and total release of Ag^+^ from the DATNFC@Ag in water at 37°C for 32 days. **(C)** Schematic illustration of the preparation of NFC, DATNFC, and DATNFC@Ag composites. Reproduced from (Li et al., 2019) with permission.

AgNPs have received a great deal of attention for their excellent antibacterial activities (Morones et al., 2005; Liu et al., 2022). The powerful antibacterial activity of AgNPs is mainly due to the release of silver ions (Ag^+^). Xiu et al. (2012) studied the interactions between AgNPs and bacterial cells. Their work suggests that antimicrobial activity can be controlled by modulating the release of Ag^+^ (Figure 8A). These possible modifications mainly include the manipulation of oxygen availability, particle size, shape, and/or type of coating. In 2018, Li et al. (2019) developed new materials which combined dialdehyde nano fibrillated cellulose and AgNPs, and Ag^+^ can be released controllably for a long time (Figure 8C). The cumulative released Ag^+^ achieved 10.06% in 32 days and Ag^+^ would be completely in 323 days (Figure 8B) to achieve long-term effective antibacterial.

Copper nanoparticles (CuNPs) presents a broad-spectrum of antibacterial activity. Over the past 2 years CuNPs has made great attention because of its remarkable antibacterial activity. Compared to AgNPs, CuNPs are more widely used in antimicrobial therapy because they are essential elements for living organisms and are more cost-effective. The set free of copper ions Cu^2+^ and ROS have been suggested as possible antibacterial mechanisms for CuNPs. Cu^2+^ are also toxic to bacterial cells. Longano et al. (2012) has produced a completely new nanocomposite, prepared by embedding laser-fabricated nano-Cu in a concentration of biodegradable polylactic acid polymers. This nanocomposite has both antimicrobial activity and biocompatibility. It shows that this CuNPs can be used in food packaging as a kind of additive. According to the past study, the silver-copper (Ag-Cu) alloy’s antibacterial activity is better than that of the metal nanoparticles alone. Jin et al. (2023) successfully used a simple chemical reduction method to prepare a kind of Ag-Cu alloy nanomaterials named Ag-CuNCs/HNT-b, and they also demonstrated that this kind of nanomaterials have good antibacterial activity. Inhibition zone testing and growth inhibition assays were used to evaluate the materials’ antibacterial capabilities, and the results revealed that the alloy materials displayed synergistic effects and increased antibacterial qualities from 30% to 53%. And it exhibited complete bactericidal behaviors.

In addition to the metal-based nanoparticles mentioned above, AuNPs are significantly more inert and biocompatible than AgNPs and CuNPs, making them potentially more effective therapeutic antibacterial agents. Although the majority of antibacterial metal or metal oxide-based nanoparticles cause cellular death primarily through the generation of ROS, the antimicrobial activity of AuNPs does not generate any ROS-related processes (Cui et al., 2012). Cui et al. (2012) found that AuNPs exhibit their antibacterial activity in two main ways: firstly, by collapsing the membrane potential and inhibiting the activity of ATPase, causing a decrease in ATP levels; secondly, by inhibiting the subunit of the ribosome from binding tRNA. Wang et al. (2021a) reported a kind of AuNPs which is co-modified by aminophenyl boronic acid and mercapto phenylboronic acid (A/M-AuNPs) showed potent and tunable antibacterial activity. As an ideal nanomaterial, it showed an extremely high median lethal dose, which is about 100 times its effective dose. Additionally, A/M-AuNPs are capable of destroying bacteria and bacteria-formed structures, such as biofilms.

Apart from these two possible mechanisms, AuNPs, also known as “light-directed nano heaters,” can effectively kill bacteria by converting the absorbed light energy into heat energy through a non-radiative decay process (Ray et al., 2012). Xu et al. developed a strategy of combining photothermal therapy (PTT) and antifouling in a single platform for antibacterial applications. Under the excitation of 808 nm near-infrared light, the surface of Au@TANPs has a 28°C temperature increase, which mean it has a killing effect on a variety of bacteria. This research offered a future strategy for very effective anti-infection medical devices. To defend against the severe risks caused by biofilms, Hu et al. (2017) reported a kind of surface-adaptive mixed charged zwitterionic 14 nm AuNPs through a simple preparation method exhibiting an enhanced photothermal ablation of *MRSA* biofilm under near-infrared (NIR) light irradiation. Besides that, these AuNPs showed no damage to the healthy tissues around the biofilm and they might be found potential application in healthcare as useful antibacterial agents.

Among the metal oxide-based nanoparticles, ZnO nanoparticles (ZnONPs) have gain more attentions than others due to their attractive properties such as good antibacterial activity, environmental friendliness, low cost, and high surface area and volume ratio (Mahamuni-Badiger et al., 2020). Jones et al. (2008) determined the antibacterial activities of six metal oxide-based nanoparticles against *S. aureus*, including ZnO, CuO, TiO_2_, Al_2_O_3_, Mg_2_O, CeO_2_. Compared to other metal oxide-based nanoparticles, ZnO showed the most significant growth inhibition and had a special antibacterial activity under visible light. To further improve the antibacterial activity and biosafety of ZnONPs, surface modification is the most effective and commonly used method. Bharathi et al. (2019) prepared a chitosan coated zinc oxide nanocomposite containing rutin (CS-ZnO). *In vitro* antibacterial experiments have shown that the CS-ZnO nanocomposite exhibited better antibacterial activity against a variety of bacteria compared to flavone rutin. Implant related infections in surgical procedures are a very difficult problem in clinical practice. Loading zinc oxide nanomaterials as a coating on the surface of medical devices may help solve such infections. Wang et al. sputtered ZnONPs onto a titanium substrate to form a uniform nano film covering material for soft tissue implantation in mice (Wang et al., 2017). The material has certain antibacterial activity *in vitro*. In the treatment of mouse soft tissue infection models, in addition to the inherent antibacterial activity of ZnONPs, zinc can also activate immune responses and further enhance antibacterial activity. It provides a new idea for the clinical application of ZnONPs.

Compared to traditional antibiotics, metal/metal oxide-based nanoparticles undoubtedly have stronger and broader antibacterial activity due to their special antibacterial mechanisms and physicochemical properties. However, due to their small size, metal/metal oxide-based nanoparticles have poor stability and are prone to aggregation, while also having the disadvantage of strong toxicity. However, the surface of metal/metal oxide-based nanoparticles is easy to modify and functionalize, and using this advantage is expected to overcome the above problems.

#### 3.3.2 Polymeric nanoparticles

Polymeric nanomaterials, due to their advantages of low toxicity, good biocompatibility, and easy degradation, more and more researchers are interested in the construction of antibacterial agents based on polymer nanoparticles (Lam et al., 2018). Polymeric nanomaterials come from a variety of sources such as natural sources, artificial sources et al. and they exhibit different characteristics. The purpose of this section is to provide an up-to-date review of the most recent studies investigating polymeric nanoparticles developed for antibacterial therapy.

Two naturally occurring polymers are proteins and carbohydrates, and they have been widely used in the preparation of polymeric nanomaterials (Ojha et al., 2018). Natural polymeric nanoparticles differ from other polymeric nanoparticles in several special ways, including biocompatibility, non-immunogenicity, and non-toxicity. Govender and colleagues create new pH-responsive nano-antibiotics (Kalhapure et al., 2017). They create a novel anionic gemini surfactant, which may be used to create pH-responsive chitosan nanoparticles (CSNPs). In order to treat *S. aureus* and *MRSA* infections, they have successfully created vancomycin-loaded pH-responsive chitosan nanoparticles that release their payload at an acidic pH. Hussain et al. (2022) used biocompatible and biodegradable polymer β-cyclodextrin to load pharmacologically active but poorly water-soluble naringin to construct polymeric nanoparticles to improve the bactericidal potential of naringin. The results of this study suggest that naringin’s-cyclodextrin nanoparticles could be employed as an effective formulation for developing a medication that can boost its bactericidal capability by destabilizing bacterial cells’ outer membranes.

By imitating the chemical structure of AMPs, the synthesized polymeric nanoparticles can acquire intrinsic antimicrobial effects by adding hydrophobic and positively charged groups to the polymer chain. The cationic moiety enables electrostatic adsorption of the polymer with negatively charged bacterial surfaces, while the hydrophobic moiety increases the membrane penetration properties (Li et al., 2012). Liu et al. (2009) found a novel class of polymeric nanoparticles created by the self-assembly of an amphiphilic peptide have stronger antimicrobial properties. When combined, these nanoparticles penetrate the blood-brain barrier, providing prospective antibacterial treatments for infections of the brain and other infectious illnesses.

With the rapid development of biological and nanoparticle technologies, more progress has been made in polymeric micelles (PMs) as carrier systems. Core-shell structure polymeric micelles could be prepared from various biodegradable and biocompatible polymers through a self-assembly process and they are usually used to improve the physicochemical and pharmacokinetic properties of drugs. Polymer micelles are usually formed by the self-assembly of individual polymer molecules, which are synthetic amphiphilic di- or tri-block copolymers consisting of hydrophilic and hydrophobic blocks. Landis et al. (2017) reported oil-in-water cross-linked polymeric micelles (∼250 nm) incorporating carvacrol oil that penetrates and eradicates multidrug-resistant (MDR) biofilms. Huang et al. (2017) proposed a combinatorial strategy to improve antibacterial efficiency, they designed a new polymer micelle for the simultaneous modification of AgNPs and encapsulation of curcumin. It could improve the water solubility of curcumin while preventing the aggregation of AgNPs, and the combined antibacterial strategy had a better antibacterial effect than a single antibacterial strategy. In addition, polymer micelles also had better biocompatibility and low hemolytic activity.

In summary, polymeric nanoparticles not only have good biocompatibility and structural stability, but also can load antibiotics, photosensitizers and other materials to further improve their antibacterial activity. In addition, it can also be applied to the surface of medical devices or mixed with some medical dressings to prevent infection and promote wound healing. This will make polymeric nanomaterials become a promising antimicrobial agent for clinical development.

#### 3.3.3 Other nanomaterials

Liposomes are vesicles composed of multiple phospholipid bilayers. They are one of the most popular organic nanoparticles for drug delivery due to their similar structure to the cell membrane and their ability to fuse with membrane structure (Forier et al., 2014). Due to their good biocompatibility, liposomes can penetrate biological membranes well and the entrapped drugs can be released into cell membranes or the interior of microorganisms, thus exhibiting some antibacterial effects (Koo et al., 2017). It has been reported that antibiotics in liposomal encapsulation led to clinically relevant biofilm formation with reduced minimum inhibitory concentration and minimum biofilm inhibitory concentration compared to free antibiotics *in vitro* (Forier et al., 2014).

Many drugs have good antibacterial and anti-biofilm pharmacological properties, but these drugs often have difficulty in achieving their efficacy due to their limited ability to penetrate cell membranes, but penetration of cells can be achieved through nanomaterials and increased drug-carrying capacity to alleviate this problem (Bandara et al., 2016). Delivery of antibiotics via liposome encapsulation is a promising antimicrobial therapy. Taking advantage of the high safety and strong targeting ability of liposomes, the physical properties of different liposomes are manipulated to design liposomes that facilitate pharmacokinetic and pharmacodynamic drug delivery (Rukavina et al., 2018).

In an example of nanomaterial-based treatment of intracellular infections, where *Salmonella* can survive and replicate within host cells and where antimicrobial therapy is also made extremely difficult by the intracellular localization of bacteria, it has been reported that enrofloxacin-loaded solid lipid nanoparticles of docosanoic acid increased the intracellular accumulation of enrofloxacin by approximately 40-fold and enhanced the killing of *Salmonella* within macrophages (Xie et al., 2017). (+)-Uric acid (2,6-diacetyl-7,9-dihydroxy-8,9b-dimethyl-1,3(2H,9bH)-dibenzofuran dione, UA) also hinders its clinical application due to its low water solubility and dose closely associated with hepatotoxicity, but when combined with suitable liposomal nanoparticles UA can control its pharmacokinetics and biodistribution while reducing side effects to improve efficacy (Francolini et al., 2019). It was found that liposomes could effectively protect cinnamaldehyde, and encapsulating cinnamon essential oil and cinnamaldehyde in liposomes could effectively delay the release rate of essential oil and improve the antibacterial ability, thus encapsulating cinnamaldehyde with liposomes both preserved its functional activity and improved its stability during processing and storage (Wang et al., 2021b).

In addition, because bacteria have difficulty surviving from physically destroyed cell structures, cationic liposomes can also destroy bacterial membranes and walls through electrostatic interactions, so cationic liposomes have specific antibacterial mechanisms and antibacterial activity, and this specific mechanism also effectively reduces the risk of enhanced bacterial resistance (Zhang et al., 2019). Cationic liposomes, such as those composed of dioctyl-dimethylammonium bromide (DODAB), are inherently inhibitory to both gram-negative and gram-positive bacteria, even without encapsulated antibiotics (Laune et al., 2022).

It is generally accepted that nanoemulsions are a mixture of water, oil, surfactants, and co-surfactants. It has received a lot of attention within the field of food antimicrobials because of its high optical transparency, thermodynamic stability, targeting action, and biocompatibility as a drug carrier (Guo et al., 2020). Essential oils, as naturally potent antimicrobial agents, can kill bacteria by their phenolic compounds reacting with the lipids in microbial membranes and releasing bacterial contents. However, the strong reactivity and low water solubility of most essential oils limit their use (Yang et al., 2021). In most cases, essential oils require specific carriers for delivery to disperse them evenly and improve their stability, and nanoemulsion delivery systems are widely used in food antimicrobial applications. The stability, as well as the antimicrobial activity of essential oils, can be improved by purifying them and then nano-emulsifying them by ultrasonication, high-pressure homogenization, and microspray methods (Prakash et al., 2019; Pongsumpun et al., 2020).

In general, essential oils have a stronger and broader antibacterial spectrum in nanoemulsions than in the free form (Yazgan et al., 2019; Ozogul et al., 2020). The nature of the nanoemulsion itself as a drug carrier, e.g., formulation, droplet size, and surface charge, can also have an impact on the nanoemulsion’s antibacterial activity (Mazarei and Rafati, 2019). Many scientific researchers have worked on the nano-emulsification of natural essential oils to improve their antimicrobial efficacy and stability. Blending co-surfactants in nanoemulsions to improve the stability of essential oils in nanoemulsions has become a research trend. Lee et al. (2019b) blended hydroxypropyl methylcellulose (HPMC) with oregano essential oil nanoemulsion to obtain a hybrid film with better performance. In addition, the antibacterial activity assay showed that the composite membrane showed good inhibition of *Salmonella typhimurium*. Other co-surfactants that played the same role are gelatin and soy lecithin, which Li et al. blended into thyme to prepare nanoemulsions that increased water vapor transmission and demonstrated broad-spectrum antibacterial activity (Li et al., 2020a). Using the same approach, Farahani et al. (2020) prepared a nanoemulsion of mixed materials with cellulose acetate nanofibres, gelatine, and wild lily, which gave smooth and homogeneous fibers after loading on the nanofilm. Results in rats showed that this fiber accelerated the wound healing process, reduced the relative wound area, and provided a good strategy for the production of wound dressings. The addition of organic phase mixtures to nanoemulsions can effectively improve the affinity of nanoemulsions for the oil phase, thus controlling the long-term stability of the nanoemulsions.

It is well known that the level of antimicrobial capacity of nanoemulsions depends mainly on the type of essential oils in them and the ratio of various substances in the emulsion (Almasi et al., 2020). And the development of nanoemulsions for food preservation has gained momentum over time. However, as far as the current research progress and applications on nanoemulsions are concerned, there are still some challenges in using nanoemulsions for antimicrobial purposes, such as masking the odor of essential oil compounds, preventing the adsorption of emulsions on food products resulting in reduced antimicrobial activity, food safety issues caused by nanoemulsions, and improving reactivity, which are challenges that deserve close attention and urgent solutions.

Microneedles have multiple micron-sized needle tips in an array loaded on a base, and by adjusting the height and shape of the loaded tips, the drug can be delivered in a targeted manner to exert its pharmacological effects. Microneedles are a rapidly developing antimicrobial patch with highly effective skin penetration. The skin is punctured by microneedles to form an orifice, after which the drug can penetrate the skin due to passive transport, effectively delivering the drug to the wound site and promoting wound healing (Frydman et al., 2020). Microneedle-delivered drugs are currently widely used in vaccines and have a very promising future in areas such as oncology and ophthalmic diseases (García et al., 2018).

As microneedle penetration depth can only reach the stratum corneum and not the inner nerve layer, making it painless and minimally invasive, safe, and efficient (Ren et al., 2020), it has rapidly attracted the attention of most scientists and gained rapid development. The modification of microneedles and their substrate materials is a proven strategy to enhance microneedle drug delivery (Su et al., 2020; Deng et al., 2022). Shi et al. (2023) loaded platinum ruthenium nanoalloy modified nanosheets onto hyaluronic acid-based microneedles and applied nanozyme antibacterial therapy, which showed good antibacterial and anti-inflammatory properties. González García et al. (2019) prepared carboxymethyl cellulose based microneedles containing AgNPs, and the results showed that they had significant antibacterial activity against both gram negative and positive bacteria. Chi et al. (2020) changed the material used for microneedle preparation to an antibacterial substance (biomass chitosan) and hydrogel as the material for the microneedle substrate, which in combination with the microneedle structure works synergistically to promote selective drug delivery. A similar approach was used by Zhang et al. (2020) and experiments in a rat model of osteoarthritis showed that microneedle patches performed well in terms of sustained drug release. In addition to the above strategies, microneedles can be encapsulated using nanofillers or some polymer films (Gao et al., 2021), thus increasing their mechanical strength and making them less likely to fracture during delivery.

The use of microneedles as a drug delivery system allows for the preservation of unstable drugs as well as their delivery. More interestingly, the use of microneedles can reduce the pain associated with traditional needle delivery of drugs as well as the risk of infection (Gonzalez-Vazquez et al., 2017). Of course, there are some limitations to the use of microneedles. The main factors currently limiting their widespread use are low adhesion and low antimicrobial activity, and many researchers have chosen to improve the material at the tip of microneedles by chemically modifying or providing microchannels to increase their adhesion (Yao et al., 2021). The antimicrobial activity is increased by loading it with antimicrobial substances such as nanoparticles, antimicrobial drugs, and metal oxides. There also needs to be some control over the exact dose size of the drug delivery and designing smart delivery platforms to rationally regulate the dose needs to be done. There is reason to believe that microneedle patches could provide a good treatment strategy for deep-seated multi-species skin infections in the future.

## 4 Summary and perspective

In this review, recent advances in the use of cationic polymers and nanomaterials for fighting bacteria and their biofilm infections are summarized. Although these nanomaterials have all made good research progress in the field of antimicrobial activity, they are still many challenges away from practical application.

(1) Biocompatibility and selectivity: These still are the most important challenge. Poor biocompatibility (toxicity of the materials themselves) and poor target selectivity are still prevalent in most of systems, and new strategies are needed to meet the stringent criteria in clinical settings.
(2) Mechanistic understanding: Most work has focused on determining the final antimicrobial agents’ outcome, but more in-depth studies using biological antimicrobial mechanisms are of great interest to help advance our understanding of bacterial-antimicrobial agent interactions and rationalize the design of new and more effective antimicrobial agents.
(3) Nanomaterial construction and antimicrobial efficiency: The antimicrobial performance of nanomaterials depends on the components and construction forms of the materials, and it is difficult to meet the practical application requirements with complex antimicrobial systems or lower antimicrobial effects.
(4) Animal experimental models: *In vitro* biological experiments provide information about the fast and efficient antimicrobial efficacy of nanomaterials; however, the results of *in vitro* experiments do not directly reflect the real effect of nanomaterials *in vivo*, such as metabolism, biodistribution, organ clearance efficiency, biodegradation, and immune responses at the organ and systemic levels, toxic response, inflammatory manifestation situation, etc.
(5) Clinical translational research: The design and development of a wide range of antimicrobial agents are most often aimed at achieving practical clinical applications, and clinical translational research on antimicrobial agents requires the concerted efforts of multidisciplinary experts.

Despite the challenges, antimicrobial materials, including cationic polymers and nanomaterials, are emerging as new tools in modern medicine. Among them, polymeric nanoparticles have the characteristics of good biocompatibility and high structural stability. AMPs can be coated with polymeric nanoparticles to regulate the excessive electrostatic action of AMPs to reduce toxicity. In addition, ultra small metal nanoclusters can also be dispersed in polymer nanoparticles to improve their poor stability and easy aggregation. They can be made into medical device coatings or wound dressings, which may have great prospects in clinical anti-infective applications. Therefore, the development and application of novel antimicrobial materials are of great importance.
